# COVID-19, body mass index and cholesterol: an ecological study using global data

**DOI:** 10.1186/s12889-021-11715-7

**Published:** 2021-09-21

**Authors:** Mohammad Sarmadi, S. Mohammad Ahmadi-Soleimani, Mohammad Fararouei, Mostafa Dianatinasab

**Affiliations:** 1 Department of Environmental Health Engineering, School of Health, Torbat Heydariyeh University of Medical Sciences, Torbat Heydariyeh, Iran; 2 Health Sciences Research Center, Torbat Heydariyeh University of Medical Sciences, Torbat Heydariyeh, Iran; 3 Department of Physiology, School of Paramedical Sciences, Torbat Heydariyeh University of Medical Sciences, Torbat Heydariyeh, Iran; 4 Neuroscience Research Center, Torbat Heydariyeh University of Medical Sciences, Torbat Heydariyeh, Iran; 5 Department of Epidemiology, Shiraz University of Medical Sciences, Shiraz, Iran; 6 Department of Complex Genetics and Epidemiology, School of Nutrition and Translational Research in Metabolism, Maastricht University, Maastricht, The Netherlands

**Keywords:** COVID-19; mortality, BMI, Obesity, Cholesterol, Risk factors

## Abstract

**Background:**

Coronavirus disease 2019 (COVID-19) is now globally considered a serious economic, social and health threat. A wide range of health related factors including Body Mass Index (BMI) is reported to be associated with the disease. In the present study, we analyzed global databases to assess the correlation of BMI and cholesterol with the risk of COVID-19.

**Methods:**

In this ecological study, we used age-standardized BMI and cholesterol levels as well as the incidence and mortality ratio of COVID-19 at the national-levels obtained from the publicly available databases such as the World Health Organization (WHO) and NCD Risk Factor Collaboration (NCD-RisC). Bivariate correlation analysis was applied to assess the correlations between the study variables. Mean differences (standard deviation: SD) of BMI and cholesterol levels of different groups were tested using independent sample t-test or Mann–Whitney rank test as appropriate. Multivariable linear regression analysis was performed to identify variables affecting the incidence and mortality ratio of COVID-19.

**Results:**

Incidence and mortality ratio of COVID-19 were significantly higher in developed (29,639.85 ± 20,210.79 for cases and 503.24 ± 414.65 for deaths) rather than developing (8153.76 ± 11,626.36 for cases and 169.95 ± 265.78 for deaths) countries (*P* < 0.01). Results indicated that the correlations of BMI and cholesterol level with COVID-19 are stronger in countries with younger population. In general, the BMI and cholesterol level were positively correlated with COVID-19 incidence ratio (β = 2396.81 and β = 30,932.80, *p* < 0.01,‌ respectively) and mortality ratio (β = 38.18 and β = 417.52, *p* < 0.05,‌ respectively) after adjusting for socioeconomic and demographic factors.

**Conclusion:**

Countries with higher BMI or cholesterol at aggregate levels had a higher ratios of COVID-19 incidence and mortality. The aggregated level of cholesterol and BMI are important risk factors for COVID-19 major outcomes, especially in developing countries with younger populations. We recommend monitoring and promotion of health indicices to better prevent morbidity and mortality of COVID-19.

**Supplementary Information:**

The online version contains supplementary material available at 10.1186/s12889-021-11715-7.

## Background

Coronavirus disease 2019 (COVID-19), is a life-threatening condition that is caused by severe acute respiratory syndrome coronavirus 2 (SARS-CoV-2) emerged first in Wuhan, China, at the end of 2019. This disease is now considered a globally severe economic, social and health issue [1]. Up to now, COVID-19 has infected approximately 180 million people worldwide, and the deaths due to the infection surpassed 3.9 million cases by June 27, 2021 [2].

World Health Organization (WHO) has declared COVID-19 a global pandemic associated with multiple affecting factors [3, 4] such as air pollution, sociodemographic indixes and genetic factors [5–10]. Previous studies suggested that Body Mass Index (BMI) and obesity are positively correlated with infection, mortality, and hospitalization due to COVID-19 [11–14]. The association of obesity with hospitalization, duration of the use of mechanical ventilation and death has been reported for other respiratory viruses, such as influenza [15, 16]. Based on the previous studies, being in a younger age is associated with a remarkably lower risk of severe COVID-19 infection [17, 18]. Furthermore, evidence indicates that young and obese individuals hospitalized with COVID- 19 diagnosis are at a higher risk for adverse outcomes [13].

On the other hand, several studies reported a lower cholesterol level in severe or end-stage COVID-19 patients than those in moderate-condition [19–23]. Currently, it is well established that BMI and serum cholesterol level have a direct correlation with the severity of COVID-19 infection [24]. However, the possible interaction between obesity, cholesterol level and age has not been fully addressed yet. Understanding COVID-19 risk factors would help both health care providers and policy makers to focus on targeted preventive and therapeutic strategies [25, 26]. In this regard, we hypothesized that a population with a higher cholesterol level and a higher rate of obesity might experience higher COVID-19 incidence and mortality (COVID-19 variables), especially in countries with a low median age (< 20 years old). To address this hypothesis, using an ecological approach, we investigated the correlation between BMI, serum cholesterol level, and COVID-19 incidence and mortality in 159 countries.

## Methods

### Study area and COVID-19 data collection

In this study, we followed the “Strengthening the Reporting of Observational Studies in Epidemiology” (STROBE) guidelines [27, 28]. We included 159 countries (each with at least 10 deaths). COVID-19 data were obtained from the free-access and publicly available World Health Organization website (WHO Coronavirus Disease Dashboard, https://covid19.who.int/). The data includes the total number of confirmed cases of COVID-19 and the death toll, along with the incidence and mortality ratios per million population by December 21, 2020. The mentioned data is updated on a daily base. The organization (WHO) also provides additional data on demographic variables such as median age (low< 20, average = 20–40 and > 40 years old), age > 65, age > 70, Gross National Product (GDP) per capita and population density (persons per km^2^) for every country. According to the United Nations, countries with high Human Development Index (HDI) scores (≥0.788) are regarded as developed, and otherwise as developing countries [29]. All methods were performed in accordance with the relevant guidelines and regulations.

### BMI and cholesterol assessment

The retrospective data on BMI and cholesterol level were collected from several publically available dataset such as WHO, Global Burden Of Diseases (GBD) and Non-Communicable Diseases Risk Factor Collaboration (NCD-RisC) [30–32]. The mean values of BMI (kg/m^2^) for every country is reported as the prevalence of underweight (BMI ≤18.5), overweight (BMI ≥25) and obese (BMI ≥30) among adults in 2016. Also, we extracted the mean total serum cholesterol level (mmole/L), prevalence of increased total cholesterol (≥5 mmol/L) and (≥6.2 mmol/L) for 2009. We obtained the mean values of the serum levels of total cholesterol, HDL and non-HDL cholesterol (mmol/L) for 2018 from NCD-RisC website [30]. Data is age-adjusted for males, females and both genders. Also, several risk factors such as air pollution, prevalence of insufficient physical activity, alcohol use, tobacco and unsafe water, sanitation, and handwashing were assessed in male, females and both genders according to GBD and WHO databases. We used data on the burden of tuberculosis and other respiratory infections (based on disability-adjusted life year (DALY) as confounder’s variables.

### Statistical analysis

Descriptive analyses (frequency distribution of qualitative variables, mean and standard deviation (SD) and median and interquartile range (IQR) for quantitative variables) were done for all variables. Spearman and Pearson’s correlation were used to estimate the associations between the COVID-19 related indexes (absolute number and cases and death per capita) with BMI, serum cholesterol level and other selected variables. The normality of data was checked by Kolmogorov–Smirnov test. Parametric approaches were applied for normally distributed data (e.g. Pearson’s’ correlation test) and non-parametric methods were used when data was not normally distributed (Spearman test). Also, to determine the adjusted contributions of independent variables, multivariable regression models were applied to reveal the association between COVID-19 incidence and mortality ratios per million and BMI and serum cholesterol level. To compare mean of quantitative variables in two or more groups, student T-test (or Mann–Whitney U if appropriate) and one-way ANOVA (or Kruskal-Wallis if appropriate) tests were used. A Scatter plot is provided to indicate the correlation of the mean values of the study variables. Statistical analyses were done using IBM SPSS Statistics 20 software and the graphical presentations are prepared by GraphPad Prism (version 6). Figures indicating the distribution of COVID-19 and independent variables are provided by GIS (Arc map 10.3).

### Role of the funding source

The funders of the study had no role in the study design, data collection, data analysis, data interpretation, or the writing of the report. The correspondig authors had full access to the data and had final responsibility for the decision to submit for publication.

## Results

### Description analysis

A total number of 77,364,641 confirmed cases and 1,702,596 deaths have been reported from 190 countries and territories by December 21, 2020. As aforementioned, our study evaluated 159 countries which included 77,102,624 COVID-19 cases and 1,697,759 deaths. USA, India, Brazil, Russia, France, UK and Turkey were the top seven countries with more than 2 million cases of COVID-19. Moreover, the USA, Brazil, India, Mexico, Italy, the UK and France reported the highest death toll.

**Fig. 1:**
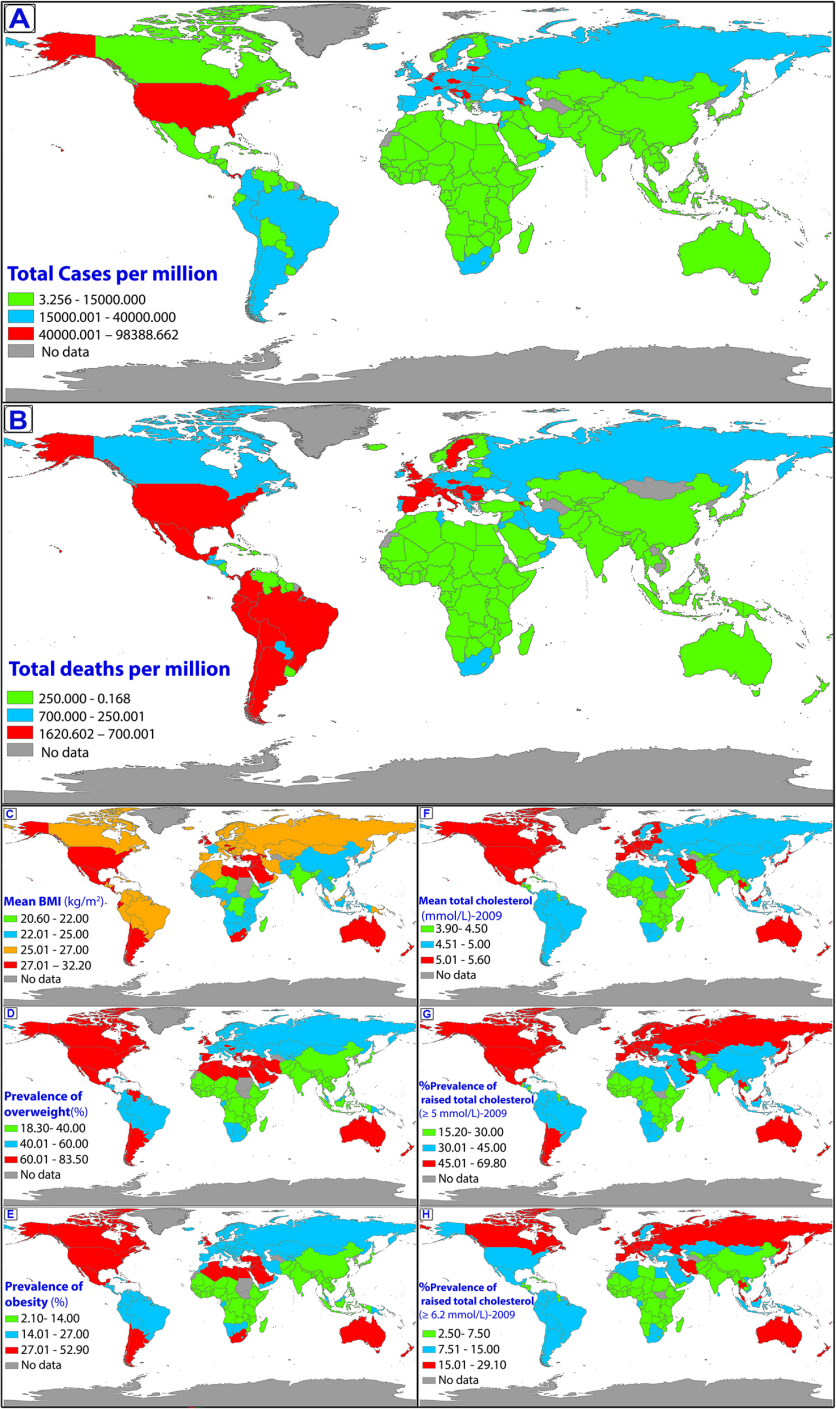
GIS distribution map of various variables; **a** cases ratio of COVID-19, **b** death ratio of COVID-19, **c** mean age-standardized BMI, **d** % prevalence of overweight (age-standardized), **e** % prevalence of obesity (age-standardized), **f** mean cholesterol level (age-standardized), **g** % prevalence of raised total cholesterol up 5 mmol/L (age-standardized) and h) % prevalence of raised total cholesterol up 6.2 mmol/L (age-standardized)

In terms of incidence ratio (per million), Andorra, Luxembourg, Montenegro, San Marino, Czechia, USA and Belgium had the greatest values. However, San Marino, Belgium, Italy, Slovenia, Bosnia and Herzegovina, Peru and Spain had the highest mortality ratios. Ethiopia and Kuwait were the countries with lowest and highest BMI, respectively, and Vietnam and Kuwait had the lowest and highest prevalence of obesity respectively. Niger and Iceland had the lowest and highest cholesterol level (mmol/L) in 2009, respectively and Rwanda and Lithuania had the lowest and highest values in 2018 of cholesterol level. The descriptive statistics for the study variables and confounding variables are shown in Table 1 and Table S1 and S2. Until December 21, 2020, the worldwide geographic distributions of the incidence and mortality ratios due to COVID-19 are shown in Fig. 1.

**Table 1:** Descriptive statistics of study

Variables	Mean	SD	Median	IQR (25, 75%)	
Total cases per million	16,396.85	18,653.61	9987.79	1106.63	27,700.01
Total deaths per million	297.82	367.55	109.60	20.32	419.96
Mean BMI (kg/m^2^)	25.55	2.00	26.10	23.80	26.90
Prevalence of underweight among adults	5.44	5.04	2.95	1.43	9.18
Prevalence of overweight among adults	47.92	15.69	55.45	30.90	60.28
Prevalence of obesity among adults	18.58	8.88	20.35	9.53	25.23
Mean total serum cholesterol-2009	4.72	.43	4.75	4.35	5.10
Prevalence of high cholesterol (≥ 5) -2009	39.90	14.36	39.40	27.30	52.95
Prevalence of high cholesterol (≥ 6.2) -2009	10.60	6.00	9.40	5.30	15.40
Mean total cholesterol (mmol/L)-2018	4.52	.38	4.62	4.15	4.81
Mean non-HDL cholesterol (mmol/L) -2018	3.28	.36	3.32	2.95	3.55
Mean HDL cholesterol (mmol/L) -2018	1.22	.16	1.19	1.11	1.38

*IQR* Interquartile Range, *BMI* Body Mass Index, *SD* Standard Deviation

### Correlation between the study variables and COVID-19 measures

Table 2 shows the Spearman and Pearson’s correlation between independent variables (BMI and cholesterol), sociodemographic factors (population density, and median age and GDP) for the selected countries with the dependent variables (COVID-19 variables).

**Table 2:** Spearman and Pearson’s correlation (r) between COVID-19 parameters and the studied variables (sex-age-standardized)

Variables	Total cases	Total deaths	Incidence ratio ^**a**^	Mortality ratio ^**a**^
Mean total serum cholesterol (2009)	.495**	.391**	.692**	.611**
Prevalence of high cholesterol (≥ 5)	.498**	.397**	.695**	.617**
Prevalence of high cholesterol (≥ 6.2)	.485**	.383**	.701**	.621**
Mean total cholesterol (mmol/L)	.450**	.409**	.609**	.602**
Mean non-HDL cholesterol (mmol/L)	.419**	.411**	.488**	.539**
Mean HDL cholesterol (mmol/L)	.199*	.139	.476**	.390**
Mean BMI (kg/m^2^)	.364**	.356**	.586**	.617**
Prevalence of overweight among adults	.438**	.410**	.641**	.643**
Prevalence of obesity among adults	.388**	.374**	.588**	.604**
Prevalence of underweight among adults	−.457**	−.436**	−.704**	−.715**
GDP per capita	.475**	.356**	.679**	.564**
Median age	.523**	.447**	.679**	.621**
Aged > 65 older	.484**	.459**	.582**	.605**
Aged > 70 older	.480**	.454**	.594**	.615**
Population density	.149	.066	.141	.033

*GDP* Gross Domestic Product per capita, ** *P* < .01, * *P* < .05, ^a^ = per million

The analysis revealed a significant and positive correlation between BMI and cholesterol with COVID-19 incidence and mortality ratios (r > 0.60 and *P* < 0.001). Althouogh, the result was not significant for the population density (*P* > .05), the association between COVID-19 indixes with BMI and serum cholesterol in males is robust (Supplementary Tables S3).

**Fig. 2:**
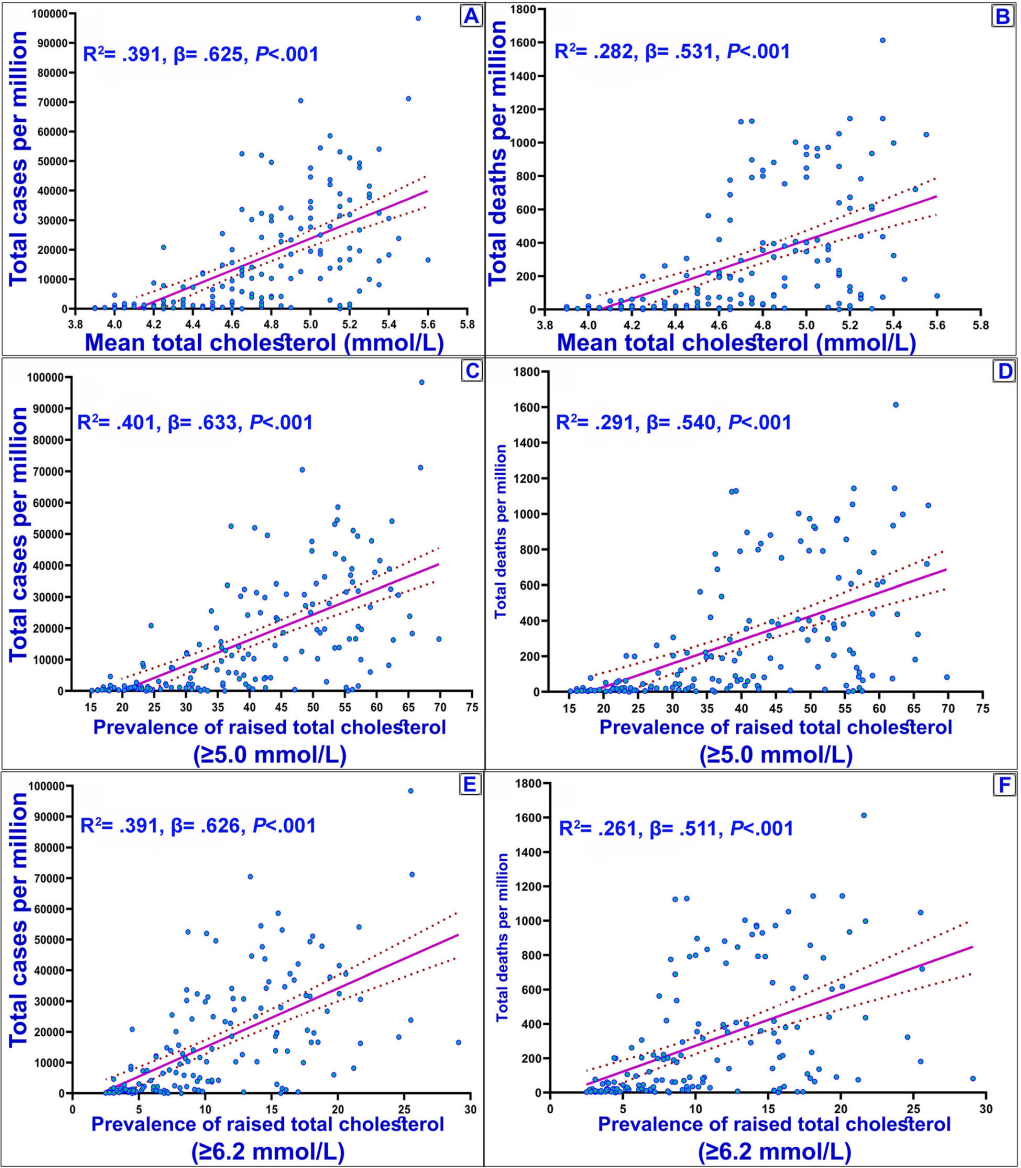
Cases and deaths ratio of COVID-19 and cholesterol variables. **a** & **b**: Mean total serum cholesterol (mmol/L) (age-standardized estimate) reported in 2009 for evaluated countries; **c** & **d**: Prevalence of raised total cholesterol (≥ 5 mmol/L or ≥ 190 mg/dl); **e** & **f**: Prevalence of raised total cholesterol (≥ 6.2 mmol/L or ≥ 240 mg/dl). Best-fit lines (solid purple line) and the 95% confidence intervals of the best-fit line (dashed) by linear regression are indicated

**Fig. 3:**
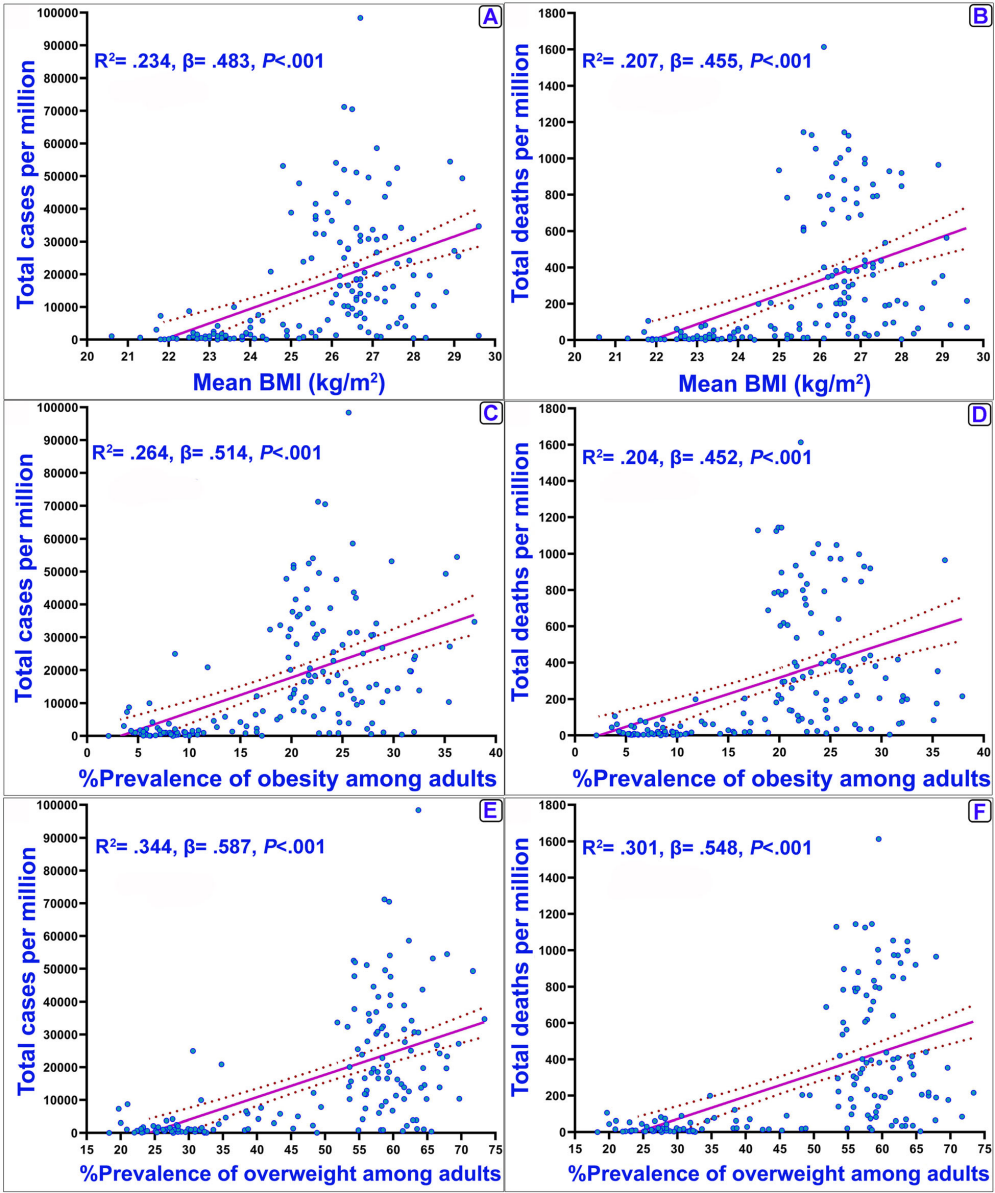
Cases and deaths ratio of COVID-19 and BMI variables. **a** & **b**: BMI in kg/m^2^ reported in 2016 for evaluated countries (age-standardized both sex); **c** & **d**: Prevalence of obesity among adults BMI ≥ 30 (age-standardized both sex); **e** & **f**: Prevalence of overweight among adults, BMI ≥ 25 (age-standardized both sex). Best-fit lines (solid purple line) and the 95% confidence intervals of the best-fit line (dashed) by linear regression are indicated

In addition, the analysis was also conducted to reveal the association between COVID-19 variables and the study variables in developed and developing countries (Supplementary Tables S4). According to the results, in developing countries, all variables have a significant and positive association with COVID-19 indixes. However, in developed countries, only the prevalence of underweight, age > 65, age > 70 and population density were significantly associated with the indixes. The associations of the independent study variables are shown in Supplementary Table S5. Accordingly, BMI had a significant positive correlation with cholesterol (r = 0.45 and r = 0.81 for BMI and Cholesterol, respectively, *P* < 0.001). The results of linear regression model also identified a positive association between the mean of total cholesterol, prevalence of raised cholesterol (≥5 mmol/L and ≥ 6.2 mmol/L) with the incidence and mortality ratios of COVID-19 (Fig. 2). These results are also observed for BMI (Fig. 3), HDL, and non-HDL cholesterol parameters (Supplementary Fig. S1).

**Fig. 4:**
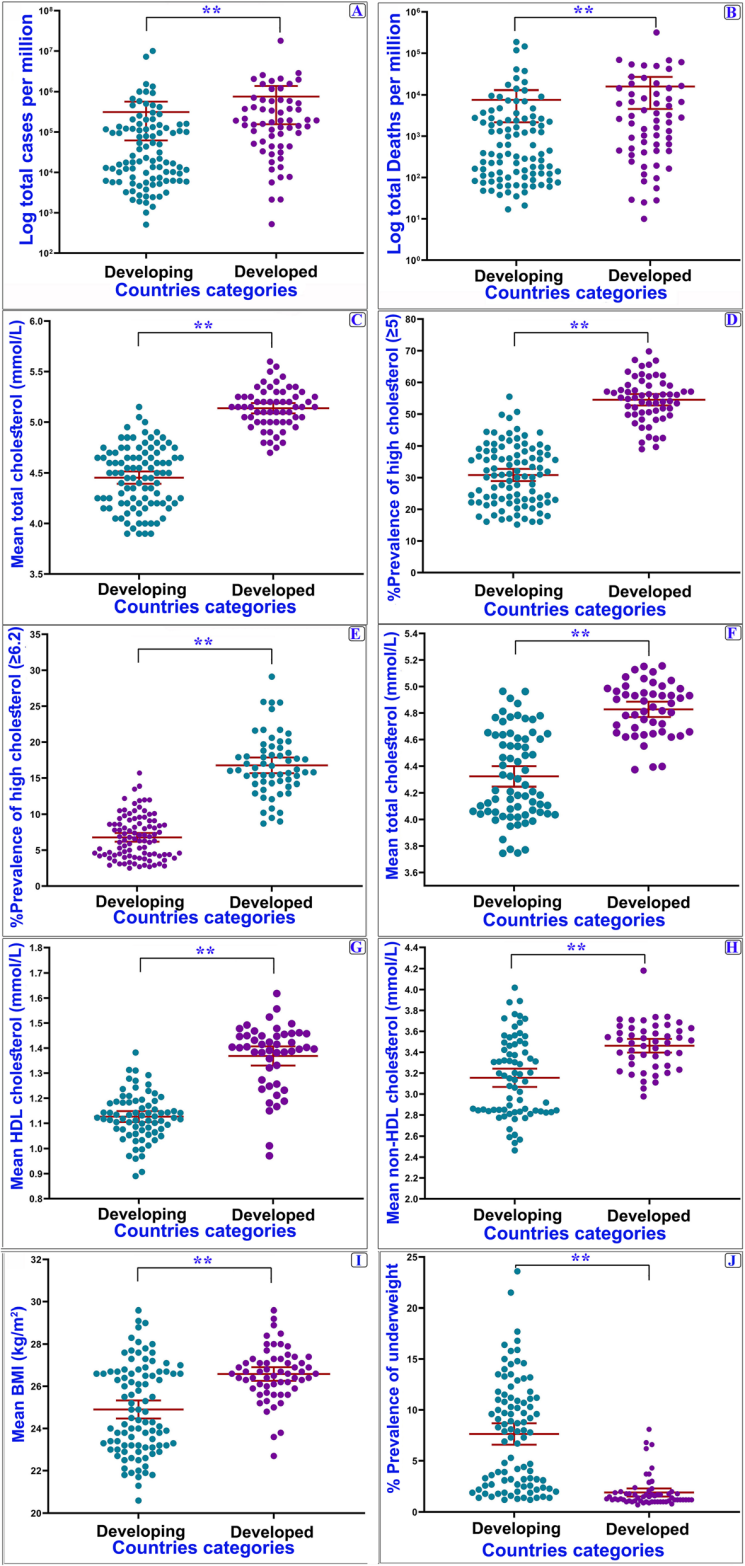
COVID-19 and independent variables in countries with different development levels. ** *P* < 0.01, Student’s t-test or Mann-Whitney U test was conducted in the comparison

**Fig. 5:**
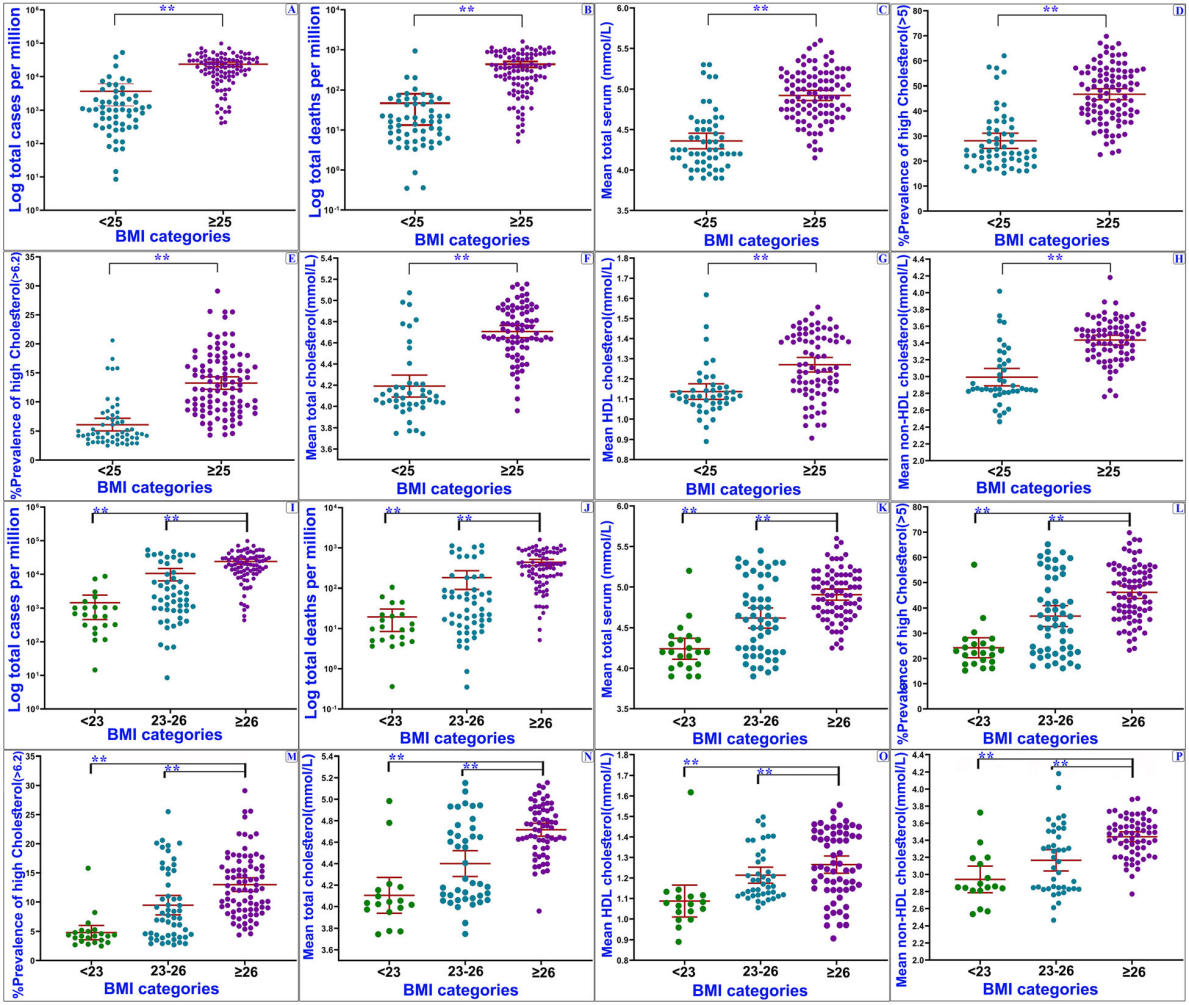
Differences of COVID-19 and independent variables in two and three BMI levels. COVID-19 cases and deaths ratio in “high BMI countries” are significantly greater than that of the “low BMI countries”. ***P < .001. Horizontal lines represent group means. One-way ANOVA analysis or Kruskal–Wallis test was conducted in the comparison

Additional analyses were performed for developed and developing countries to investigate the impact of independent variables on COVID-19 indixes. In this respect, we found significant differences between COVID-19 variables (*P* < 0.01) (Fig. 4). Among developing countries, the average mean cholesterol (2009) and BMI were 4.45 (95% Confidence Interval (95% CI): 4.39–4.51) and 24.90 (95% CI: 24.47–25.33), which was elevated with an increase in the incidence and mortality ratios of COVID-19 (*P* < 0.01). These variables were 5.14 (95% CI: 5.09–5.19) and 26.59 (95% CI: 26.26–26.91) in developed countries. There were significant differences between the countries in BMI categories (*p* < 0.001) (Fig. 5). Among countries with low (< 25 Kg/m^2^) and high (≥25 Kg/m^2^) BMI, the average incidence ratio (per million) was 3714.67 (95% CI: 1348.18–6081.14) and 23,551.94 (95% CI: 19858.26–27,245.63), and the mortality ratio was 46.93 (95% CI: 13.32–80.53) and 434.53 (95% CI: 361.60–507.46), respectively. The regions with larger BMI showed significantly higher cholesterol level and higher incidence of COVID-19 compared to the countries with low BMI (Fig. 5).

### Regression analysis

Table 3 demonstrates the results of regression analysis (adjusted and non-adjusted) on the association of the incidence and mortality ratios of COVID-19 with mean BMI and cholesterol variables.

**Table 3:** Regression coefficients^a^ for mutually adjusted associations between COVID-19 indixes and independent variables, age-sex-adjusted

Model	Unstandardized Coefficients		Standardized Coefficients	t	Sig.
	B	Std. Error	Beta (β)		
**Incidence ratio per million (no adjusted)**					
Mean BMI (kg/m^2^)	4432.45	647.12	.48	6.85	.000
Mean total cholesterol	26,843.19	2693.20	.62	9.97	.000
**Incidence ratio per million (Full Adjusted)**					
Mean BMI (kg/m^2^)	2396.81	881.54	.26	2.72	.007
Mean total cholesterol	30,932.80	8371.40	.72	3.70	.000
**Mortality ratio per million (no adjusted)**					
Mean BMI (kg/m^2^)	80.38	12.69	.46	6.33	.000
Mean total cholesterol	439.12	56.33	.53	7.80	.000
**Mortality ratio per million (Full Adjusted)**					
Mean BMI (kg/m^2^)	38.18	18.36	.22	2.08	.040
Mean total cholesterol	417.52	174.40	.50	2.39	.018

*BMI* Body Mass Index, *HDI* Human Development Index

^a^adjusted for HDI, air pollution, alcohol use, tobacco, unsafe water and sanitation, handwashing facility of respiratory infections, tuberculosis diseases and prevalence of insufficient physical activity

Results revealed a positive association between the incidence ratio (per million) and mean BMI (β = 2396.81, *p* = 0.007) and serum cholesterol level (β = 30,932.80, *p* < 0.001). Also, there were positive correlations between mortality ratio (per million) with mean BMI (β = 38.18, *p* < 0.001) and serum cholesterol level (β = 417.52, *p* < 0.001). Also, regression analysis between age groups showed a robust association between COVID-19 incidence and mortality ratios with BMI and cholesterol level in countries with a median age under 20 years compared to countries with the median age between 20 and 40 and > 40 years.

## Discussion

In the present national-based study, we examined the association between BMI, cholesterol level and COVID-19 incidence and mortality ratios using an ecological approach. In contrast to the previous studies suggesting an inverse correlation between obesity and mortality in patients with pneumonia and acute respiratory distress syndrome ARDS [33–35], we reported a strong positive correlation between BMI, cholesterol level and incidence and mortality due to COVID-19, even after adjusting for potential confounding factors such as air pollution, socioeconomic status and lifestyle (Table 3). With regard to BMI, our findings were consistent with the previous studies indicating the role of obesity in the severity and mortality of COVID-19 disease and its associated complications [11, 12, 36–39]. Furthermore, in line with the previous population-level studies [11–13, 40, 41], we observed a correlation between larger BMI and higher incidence and mortality of COVID-19 (Figs. 1 and 5). For example, in a study by Hendren et al. (2020), it was demonstrated that obesity is more common in the population of patients hospitalized for COVID-19 compared to ambulatory cases, especially, among those who are under the age of 50 years [11].

It should be noted that the applied BMI cut-offs might be different among countries and throughout the years, which in turn may affected the results. However, WHO has categorized countries based on the same defined cut-off points. Various studies have also shown that the BMI categorisations used by WHO and NCD-RisC has undergone negligible changes over decades for children and adults [42, 43]. Considering the role of age, we conducted a subgroup analysis for the age categories (Table 4). Results indicated that in countries with a lower median age, there is a stronger associations between COVID-19 indixes with BMI and cholesterol level (Table 4). Similarly, a cross-sectional study has reported a higher risk of in-hospital death or the need for mechanical ventilation in obese patients who were under 35 years of age [44]. In another study, Tartof et al., reported stronger associations between BMI and COVID-19 mortality in patients under the age of 60 (compared to younger population) regardless of being either outpatient or inpatient [13]. Obesity is also associated with intubation or death among patients younger than 65 years [12].

**Table 4:** Association between COVID-19 indixes and independent variables by median age categories

Independent variables	Median age < 20 years		Median age20–40 years		Median age > 40 years	
	R^**2**^	β	R^**2**^	β	R^**2**^	β
**Incidence ratio per million**						
Mean BMI (kg/m^2^)	.663	.814**	.141	.376**	.156	.395*
Prevalence of overweight	.768	.877**	.198	.445**	.244	.494**
Prevalence of obesity	.764	.874**	.160	.400**	.162	.403*
Mean total cholesterol mmol/L (2009)	.307	.554**	.212	.460**	.139	.373*
Prevalence of cholesterol ≥5 mmol/L(2009)	.394	.628**	.220	.469**	.133	.365*
Prevalence of cholesterol ≥6.2 mmol/L (2009)	.456	.675**	.194	.440**	.152	.389*
Mean total cholesterol (2018)	.670	.819**	.092	.303*	.045	.211
Mean non-HDL cholesterol (mmol/L) (2018)	.631	.794**	.016	.126	.031	.176
Mean HDL cholesterol (mmol/L) (2018)	.163	.403	.037	.192	.004	.063
**Mortality ratio per million**						
Mean BMI (kg/m^2^)	.675	.822**	.140	.375**	.145	.380*
Prevalence of overweight	.762	.873**	.163	.403**	.246	.496**
Prevalence of obesity	.761	.872**	.105	.324**	.147	.383*
Mean total cholesterol mmol/L (2009)	.327	.572**	.081	.284**	.047	.217
Prevalence of cholesterol ≥5 mmol/L(2009)	.423	.650**	.085	.292**	.052	.228
Prevalence of cholesterol ≥6.2 mmol/L (2009)	.488	.699**	.054	.232*	.050	.223
Mean total cholesterol (2018)	.675	.822**	.095	.308**	.001	.024
Mean non-HDL cholesterol (mmol/L) (2018)	.653	.808**	.060	.245*	.000	.003
Mean HDL cholesterol (mmol/L) (2018)	.155	.393	.004	−.063	.000	−.005

*BMI* Body Mass Index, *β* standardized coefficients for regression; **P < .001, *P < .05

The possible reason for the observed association between obesity with incidence, mortality and the need for respiratory support among younger individuals is not still clear. However, one possibility is that the weaker associations of high BMI with mortality in older individuals may reflect the fact that in older patients, there is more competing life-threatening risks such as co-morbid diseases. Thus, in younger individuals, obesity and high cholesterol levels affect COVID-19 incidence rate more significantly than older individuals.

With regard to the mechanisms underlying the association between obesity and higher risk of COVID-19 infection, first, it is currently well established that obesity is associated with increased prevalence of cardiometabolic conditions such as diabetes and hypertension, that adversely complicate the therapeutic outcomes in COVID-19 patients [13, 45, 46]. Second, the virus responsible for COVID-19 disease (SARS-CoV-2), exhibits a high affinity for angiotensin-converting enzyme 2 (ACE2) in host cells which is essential for cellular resistance to infections [47]. ACE2 is widely expressed in adipose tissue; therefore, the excessive presence of fat (as we expect in obese patients) may aggravate the severity of infections [48, 49]. Third, obesity reduces the respiratory output parameters such as compliance, expiratory reserve volume and functional capacity [50]. These may worsen the severity of COVID-19 disease. Besides, there is evidence indicating that the capability of immune system to respond effectively against an infection is adversely altered in obese individuals [51–57].

Another aspect of our study addressed the correlation between cholesterol level and COVID-19 incidence and mortality ratios. Up to now, limited studies have assessed this issue in the context of COVID-19 and most of them reported that patients with more severe viral infection display lower cholesterol levels [20, 21, 23]. As for the lipoproteins, the literature is controversial. In a retrospective study conducted in Wuhan, China, it was shown that HDL and total cholesterol levels are inversely correlated with infection severity; however, serum LDL levels were found to be higher in more severe cases [58]. Another study from Wuhan has reported significantly lower total cholesterol, HDL and LDL among infected patients [59]. In addition there are findings obtained regarding other infectious diseases in this respect. For example, in a study conducted on 3961 sepsis cases, it was found that increased LDL level is associated with a higher risk of sepsis and the need for a stay in intensive care unit [60]. Lipid metabolism is critical for the virus lifecycle processes (such as endocytosis, exocytosis, replication and membrane homeostasis) [61].

Current clinical experiences from COVID-19 infection have revealed alteration in lipid metabolism after recovery of the patients, suggesting a potential biological relevance [62]. Pharmacological reduction of cholesterol has been found to suppress a type of coronavirus responsible for infectious bronchitis by disrupting lipid rafts that normally enable the virus to enter the cells [63].

### Strengths and limitations

This is the first ecological study to evaluate the association of BMI and cholesterol level in the context of COVID-19 distribution. We believe our findings would help the researchers reach a broader and better understanding of the factors affecting COVID-19 incidene and mortality. Ecological studies are characterized with several appealing features such as reliance on anonymous (mostly public domain) data covering extensive geographical areas at both regional and national levels. This particularly matters when there is no available data at individual-level. In addition, the use of ecological data provides great advantage in terms of time and cost. Another benefit for this type of study is prevention of measurement error of individual-level such as air pollution exposure, age-gender-adjusted BMI, and alcoholic bevareage consumption [64, 65]. Moreover, ecological studies have also been conducted to assess the mortality patterns in different countries [66].

However, this work also faced certain limitations as follow: Considering the ecological nature of this study (ecological fallacy), data collection was large scale; therefore, individual values of BMI and cholesterol may not be thoroughly representative actual values. Moreover, COVID-19 variables, lifestyle and sociodemographic trends are described at the national level. As a result, discrepancies may exist among the population subgroups in each country. On the other hand, although obesity cut-off points were the same for all countries, this may not be precise for some regions, due to ethnic variations in reporting BMI [67]. It should be noted that BMI cut-offs for children and adolescents are different from adults, and due to the limited number and low incidence rate of COVID-19 in children and also the lack of categorization of COVID-19 data by age, the potential effects are not considered in this study. Ecological studies are generally regarded as the preliminary step of epidemiologic studies, focusing on disease prevalence amongst various populations [68]. Therefore, ecological correlation addressing the risk factors certainly requires further confirmation provided by longitudinal studies and randomized trials [65, 69, 70].

## Conclusion

The incidence and mortality ratios of COVID-19 showed that in some developing and developed countries, there are significant differences with regard to age, BMI and cholesterol level. This may indicate the existance of underlying factors worldwide. However, future studies are needed to verify these possibilities more precisely. The regional variability of incidence and mortality ratios observed among different countries can be partly explained by the higher prevalence of obesity and higher cholesterol level in these areas. Collectively, our results suggest the promotion of specific health indicices (including BMI and cholesterol level) is effective to reduce the risk of COVID-19 disease. This is particularly important for developing countries with low median age.

## Supplementary Information

Additional file 1: Supplementary appendix.

## Data Availability

All data generated or analysed during this study are included in this published article and publicy avalable dataset. Risk factors data analyzed in this study are available in the GBD data tool: http://ghdx.healthdata.org/gbd-results-tool. The main source of BMI and cholestero data in the world was the WHO website: https://www.euro.who.int/en/health-topics/disease-prevention/nutrition/a-healthy-lifestyle/body-mass-index-bmi. Data related to the COVID-19 variables available in https://covid19.who.int/ website.

## Abbreviations

COVID-19: Coronavirus disease 2019
BMI: Body Mass Index
NCD-RisC: NCD-Risk Factor Collaboration
WHO: World Health Organization
GDP: Gross National Product
GBD: Global Burden of Diseases
HDI: Human Developlent Index
HDL: High-Density Lipoprotein
LDL: Low-Density Lipoprotein
DALY: Disability-Adjusted Life Year
ANOVA: Analysis of Variance
UK: United Kingdom
USA: United States of America
IQR: Interquartile Range
SD: Standard Deviation
GIS: Geographic Information System
CI: Confidence Interval
ACE2: Angiotensin-Converting Enzyme 2
SARS-CoV-2: Severe Acute Respiratory Syndrome Coronavirus 2
