# Supplementary material for: COVID-19, body mass index and cholesterol: an ecological study using global data
Source: BMC Public Health. 2021 Sep 21;21:1712. doi: 10.1186/s12889-021-11715-7 (PMC8453032; doi:10.1186/s12889-021-11715-7)
Supplement: Supplementary file 1 — Supplementary appendix. [file 12889_2021_11715_MOESM1_ESM.docx]

**Supplementary appendix**

This appendix formed part of the original submission.

We post it as supplied by the authors.

Supplement to:

**COVID-19, Body Mass Index and Cholesterol: An Ecological Study Using Global Data**

Mohammad Sarmadi^1, 2*#^, S. Mohammad Ahmadi-Soleimani^3, 4*#^, Mohammad Fararouei^5^, Mostafa Dianatinasab^6^

^1^ Department of Environmental Health Engineering, School of Health, Torbat Heydariyeh University of Medical Sciences, Torbat Heydariyeh, Iran

^2^ Health Sciences Research Center, Torbat Heydariyeh University of Medical Sciences, Torbat Heydariyeh, Iran.

^3^ Department of Physiology, School of Paramedical Sciences, Torbat Heydariyeh University of Medical Sciences, Torbat Heydariyeh, Iran.

^4^Neuroscience Research Center, Torbat Heydariyeh University of Medical Sciences, Torbat Heydariyeh, Iran.

^5^ Department of Epidemiology, Shiraz University of Medical Sciences, Shiraz, Iran.

^6^ Department of Complex Genetics and Epidemiology, School of Nutrition and Translational Research in Metabolism, Maastricht University, Maastricht, The Netherlands.

**Table S1. Mean ±SD value of variables in median age and developed & developing categories**

| **Categories** | **Developing countries** | | | | | | **Developed countries** | | | | | |
| --- | --- | --- | --- | --- | --- | --- | --- | --- | --- | --- | --- | --- |
| **Median age** | **<20** | | **20-40** | | **>40** | | **<20** | | **20-40** | | **>40** | |
|  | **Mean** | **SD** | **Mean** | **SD** | **Mean** | **SD** | **Mean** | **SD** | **Mean** | **SD** | **Mean** | **SD** |
| Incidence ratio per million | 1191.33 | 2494.93 | 11147.91 | 12090.22 | 20167.35 | 19554.52 | ND | ND | 26292.41 | 20572.35 | 32480.11 | 19765.52 |
| Mortality ratio per million | 25.01 | 53.99 | 235.00 | 285.06 | 387.92 | 458.64 | ND | ND | 344.90 | 327.45 | 637.59 | 437.44 |
| Population density (km^2^) | 77.44 | 64.52 | 169.99 | 264.34 | 94.34 | 27.73 | ND | ND | 165.40 | 373.53 | 437.73 | 1368.98 |
| Median age (year) | 18.33 | 1.21 | 27.78 | 4.82 | 41.66 | 1.17 | ND | ND | 34.75 | 3.38 | 43.59 | 1.99 |
| aged >65 older | 2.90 | .38 | 6.16 | 2.62 | 15.30 | 2.40 | ND | ND | 9.78 | 5.07 | 19.04 | 2.67 |
| aged >70 older | 1.69 | .27 | 3.77 | 1.73 | 9.61 | 1.91 | ND | ND | 6.17 | 3.41 | 12.66 | 2.29 |
| GDP per capita | 2697.00 | 2682.81 | 9495.16 | 5538.59 | 12483.71 | 3582.29 | ND | ND | 40634.50 | 24752.83 | 37049.85 | 13518.49 |
| Extreme poverty | 42.79 | 18.89 | 9.07 | 12.95 | 0.13 | .06 | ND | ND | 0.56 | 0.58 | .94 | 1.22 |
| Life expectancy | 61.96 | 4.38 | 71.65 | 5.14 | 76.28 | 2.56 | ND | ND | 78.52 | 3.15 | 80.75 | 3.00 |
| HDI | .49 | .07 | .67 | .09 | .77 | .01 | ND | ND | .85 | .05 | 0.88 | .04 |
| Prevalence of overweight | 28.78 | 7.64 | 47.01 | 14.53 | 51.98 | 11.04 | ND | ND | 61.28 | 8.22 | 56.41 | 9.22 |
| Prevalence of obesity | 8.91 | 4.56 | 18.27 | 8.44 | 19.62 | 6.00 | ND | ND | 26.77 | 6.11 | 21.51 | 6.04 |
| Mean BMI (kg/m²) | 23.19 | 1.34 | 25.76 | 1.97 | 25.70 | .82 | ND | ND | 27.18 | 1.17 | 26.07 | 1.10 |
| Mean total cholesterol (2009) | 4.17 | .21 | 4.58 | .22 | 4.85 | .25 | ND | ND | 5.08 | .22 | 5.19 | .16 |
| Mean total cholesterol (2018) | 4.05 | .17 | 4.44 | .31 | 4.60 | .06 | ND | ND | 4.73 | .20 | 4.92 | .15 |
| Prevalence of high cholesterol (≥ 5) | 22.33 | 5.93 | 34.50 | 7.22 | 44.16 | 9.16 | ND | ND | 52.30 | 7.71 | 56.46 | 5.74 |
| Prevalence of high cholesterol (≥ 6.2) | 4.21 | 1.59 | 7.84 | 2.41 | 11.48 | 3.44 | ND | ND | 15.55 | 4.64 | 17.83 | 3.64 |
| Mean HDL cholesterol | 1.08 | .06 | 1.14 | .10 | 1.27 | .07 | ND | ND | 1.30 | .15 | 1.44 | .06 |
| Mean non-HDL cholesterol | 2.86 | .18 | 3.29 | .37 | 3.30 | .02 | ND | ND | 3.44 | .27 | 3.49 | .18 |
| ND: no data, SD: standard deviation; BMI: body mass index; GDP= Gross domestic product; HDI: human development index; ^a^=per million | | | | | | | | | | | | |

**Table S2. Descriptive statistics for the confounding variables**

| **Categories** | **Developing countries** | | | | | | **Developed countries** | | | | | |
| --- | --- | --- | --- | --- | --- | --- | --- | --- | --- | --- | --- | --- |
| **Median age** | **<20** | | **20-40** | | **>40** | | **<20** | | **20-40** | | **>40** | |
|  | **Mean** | **SD** | **Mean** | **SD** | **Mean** | **SD** | **Mean** | **SD** | **Mean** | **SD** | **Mean** | **SD** |
| Population density (per km2) | 77.44 | 64.52 | 169.99 | 264.34 | 94.34 | 27.73 | ND | ND | 165.40 | 373.53 | 437.73 | 1368.98 |
| Median age (year) | 18.33 | 1.21 | 27.79 | 4.82 | 41.66 | 1.17 | ND | ND | 34.75 | 3.38 | 43.59 | 1.99 |
| Aged 65 older (%) | 2.90 | 0.38 | 6.16 | 2.62 | 15.30 | 2.40 | ND | ND | 9.78 | 5.07 | 19.04 | 2.67 |
| Aged 70 older (%) | 1.69 | .27 | 3.77 | 1.73 | 9.61 | 1.91 | ND | ND | 6.17 | 3.41 | 12.66 | 2.29 |
| GDP per capita ($US) | 2697.00 | 2682.81 | 9495.16 | 5538.59 | 12483.71 | 3582.29 | ND | ND | 40634.50 | 24752.83 | 37049.85 | 13518.49 |
| Extreme poverty (%) | 42.79 | 18.89 | 9.07 | 12.95 | .13 | .06 | ND | ND | .56 | .58 | .94 | 1.22 |
| HDI | .49 | .07 | .67 | .09 | .77 | .01 | ND | ND | .85 | .05 | .88 | .04 |
| DALYs of Alcohol use | 485.73 | 377.36 | 191.14 | 326.93 | 80.25 | 81.72 | ND | ND | 30.94 | 38.16 | 36.08 | 29.73 |
| DALYs of air pollution | 1708.81 | 870.99 | 340.36 | 356.30 | 58.18 | 37.83 | ND | ND | 59.08 | 56.53 | 19.80 | 19.61 |
| DALYs of Tobacco | 545.22 | 303.58 | 302.35 | 332.47 | 168.28 | 94.87 | ND | ND | 123.74 | 111.70 | 81.20 | 52.98 |
| DALYs of unsafe water and sanitation, handwashing facility | 743.99 | 314.74 | 174.50 | 222.85 | 22.61 | 22.61 | ND | ND | 15.48 | 13.49 | 3.40 | 2.79 |
| Prevalence of insufficient of Physical Activity | 20.79 | 10.21 | 27.96 | 10.14 | 29.20 | 8.53 | ND | ND | 35.69 | 11.05 | 32.32 | 6.94 |
| SD: standard deviation; GDP= Gross domestic product; HDI: human development index; DALY: disability-adjusted life year | | | | | | | | | | | | |

**Table S3. The Spearman and Pearson’s correlation (r) between COVID-19 parameters and independent variables (cholesterol and BMI variables are age-standardized)**

| **variables** | **Total cases** | **Total deaths** | **Incidence ratio ^a^** | **mortality ratio ^a^** |
| --- | --- | --- | --- | --- |
| Mean total serum cholesterol (2009)_Male | .499** | .402** | .698** | .622** |
| Mean total serum cholesterol (2009)_female | .491** | .384** | .681** | .596** |
| Prevalence of high cholesterol (≥ 5)_Male | .498** | .402** | .701** | .626** |
| Prevalence of high cholesterol (≥ 5)_Female | .490** | .386** | .683** | .602** |
| Prevalence of high cholesterol (≥ 6.2)_Male | .486** | .387** | .705** | .626** |
| Prevalence of high cholesterol (≥ 6.2)_Female | .481** | .377** | .689** | .608** |
| Mean total cholesterol (mmol/L)_ Male | .494** | .433** | .598** | .563** |
| Mean non-HDL cholesterol (mmol/L)_ Male | .430** | .393** | .502** | .495** |
| Mean HDL cholesterol (mmol/L)_ Male | .291** | .233** | .500** | .440** |
| Mean total cholesterol (mmol/L)_ female | .423** | .366** | .581** | .545** |
| Mean non-HDL cholesterol (mmol/L)_ female | .298** | .286** | .348** | .365** |
| Mean HDL cholesterol (mmol/L)_ female | .332** | .255** | .571** | .484** |
| Mean BMI (kg/m²)_ Male | .436** | .403** | .681** | .666** |
| Mean BMI (kg/m²)_ Female | .262** | .267** | .458** | .514** |
| Prevalence of overweight among adults_ Male | .511** | .463** | .747** | .717** |
| Prevalence of overweight among adults_ Female | .319** | .326** | .492** | .544** |
| Prevalence of obesity among adults_ Male | .469** | .422** | .709** | .676** |
| Prevalence of obesity among adults_ Female | .290** | .291** | .474** | .516** |
| Prevalence of underweight among adults_ Male | -.475** | -.450** | -.733** | -.733** |
| Prevalence of underweight among adults_ Female | -.437** | -.431** | -.652** | -.693** |
| GDP per capita | .475** | .356** | .679** | .564** |
| Median age | .523** | .447** | .679** | .621** |
| Aged >65 older | .484** | .459** | .582** | .605** |
| Aged >70 older | .480** | .454** | .594** | .615** |
| Population density (person per KM2) | .149 | .066 | .141 | .033 |
| BMI: body mass index; GDP= Gross domestic product; **P< .001; ^a^=per million | | | | |

**Table S4. The Spearman and Pearson’s correlation (r) between COVID-19 ratio (per million) and independent variables include cholesterol and BMI variables (for both sex and age-standardized) in developed and developing countries.**

| variable | **Developing** | | **Developed** | |
| --- | --- | --- | --- | --- |
|  | **Incidence ratio ^a^** | **mortality ratio ^a^** | **Incidence ratio ^a^** | **mortality ratio ^a^** |
| Mean total cholesterol (2009)_Male | .620** | .629** | .179 | .130 |
| Mean total cholesterol (2009)_female | .613** | .620** | .103 | _.002 |
| Mean total cholesterol (2009) _Mean | .622** | .628** | .133 | .054 |
| Prevalence of high cholesterol (≥ 5)_Male | .622** | .628** | .203 | .163 |
| Prevalence of high cholesterol (≥ 5)_Female | .610** | .617** | .114 | .036 |
| Prevalence of high cholesterol (≥ 5)_both sex | .621** | .628** | .165 | .105 |
| Prevalence of high cholesterol (≥ 6.2)_Male | .616** | .618** | .222 | .163 |
| Prevalence of high cholesterol (≥ 6.2)_Female | .616** | .616** | .079 | .021 |
| Prevalence of high cholesterol (≥ 6.2)_both sex | .625** | .625** | .157 | .107 |
| Mean total cholesterol (mmol/L) _Male | .564** | .592** | .024 | .084 |
| Mean total cholesterol (mmol/L) _Female | .478** | .524** | .093 | .111 |
| Mean total cholesterol (mmol/L) _both sex | .623** | .677** | .116 | .175 |
| Mean HDL cholesterol (mmol/L) _Male | .333** | .287** | .019 | .171 |
| Mean HDL cholesterol (mmol/L) _Female | .395** | .338** | .086 | .188 |
| Mean HDL cholesterol (mmol/L) _both sex | .344** | .250* | -.026 | .063 |
| Mean non-HDL cholesterol (mmol/L) _Male | .489** | .540** | .030 | .037 |
| Mean non-HDL cholesterol (mmol/L) _Female | .350** | .420** | .012 | -.030 |
| Mean non-HDL cholesterol (mmol/L)_both sex | .507** | .595** | .162 | .176 |
| Mean BMI (kg/m²)_Male | .649** | .699** | .143 | .135 |
| Mean BMI (kg/m²)_Female | .640** | .696** | -.046 | -.017 |
| Mean BMI (kg/m²)_both sex | .658** | .713** | .052 | .056 |
| Prevalence of overweight among adults_ Male | .674** | .723** | .312* | .309* |
| Prevalence of overweight among adults_ Female | .541** | .620** | .122 | .057 |
| Prevalence of overweight among adults_ both sex | .600** | .668** | .195 | .137 |
| Prevalence of obesity among adults_ Male | .641** | .693** | .195 | .139 |
| Prevalence of obesity among adults_ Female | .525** | .596** | .085 | .027 |
| Prevalence of obesity among adults_ both sex | .586** | .656** | .105 | .055 |
| Prevalence of underweight among adults_ Male | -.617** | -.670** | -.297* | -.436** |
| Prevalence of underweight among adults_ Female | -.604** | -.669** | -.172 | -.239 |
| Prevalence of underweight among adults_ both sex | -.606** | -.669** | -.220 | -.311* |
| GDP per capita | .644** | .590** | .093 | -.149 |
| Median age | .641** | .597** | .097 | .305* |
| Aged >65 older | .608** | .585** | .089 | .347** |
| Aged >70 older | .616** | .597** | .114 | .362** |
| Population density(person per KM^2^) | .021 | -.033 | .333** | .123 |
| BMI: body mass index; GDP= Gross domestic product; **P< .001, *P< .05, ^a^=per million | | | | |

**Table S5. The Spearman and Pearson’s correlation (r) between independent variables.**

| Variables | **1.** | **2.** | **3.** | **4.** | **5.** | **6.** | **7.** | **8.** | **9.** | **10** |
| --- | --- | --- | --- | --- | --- | --- | --- | --- | --- | --- |
| 1.Mean BMI | 1.000 |  |  |  |  |  |  |  |  |  |
| 2.Prevalence of overweight | .849** | 1.000 |  |  |  |  |  |  |  |  |
| 3.Prevalence of obesity | .873** | .969** | 1.000 |  |  |  |  |  |  |  |
| 4.Prevalence of underweight | -.777** | -.855** | -.786** | 1.000 |  |  |  |  |  |  |
| 5.Mean total cholesterol (2009) | .519** | .669** | .572** | -.754** | 1.000 |  |  |  |  |  |
| 6.Mean total cholesterol (2018) | .509** | .579** | .510** | -.694** | .772** | 1.000 |  |  |  |  |
| 7.Prevalence of high cholesterol (≥ 5) | .520** | .667** | .572** | -.758** | .996** | .769** | 1.000 |  |  |  |
| 8.Prevalence of high cholesterol (≥ 6.2) | .525** | .665** | .575** | -.760** | .965** | .712** | .977** | 1.000 |  |  |
| 9.Mean HDL cholesterol | .324** | .467** | .410** | -.601** | .751** | .696** | .770** | .789** | 1.000 |  |
| 10.Mean non-HDL cholesterol | .509** | .494** | .461** | -.525** | .555** | .910** | .539** | .460** | .388** |  |
| 11.Median age | .449** | .557** | .468** | -.714** | .848** | .810** | .849** | .851** | .772** | .582** |
| BMI: body mass index; **P< .001 | | | | | | | | | |  |


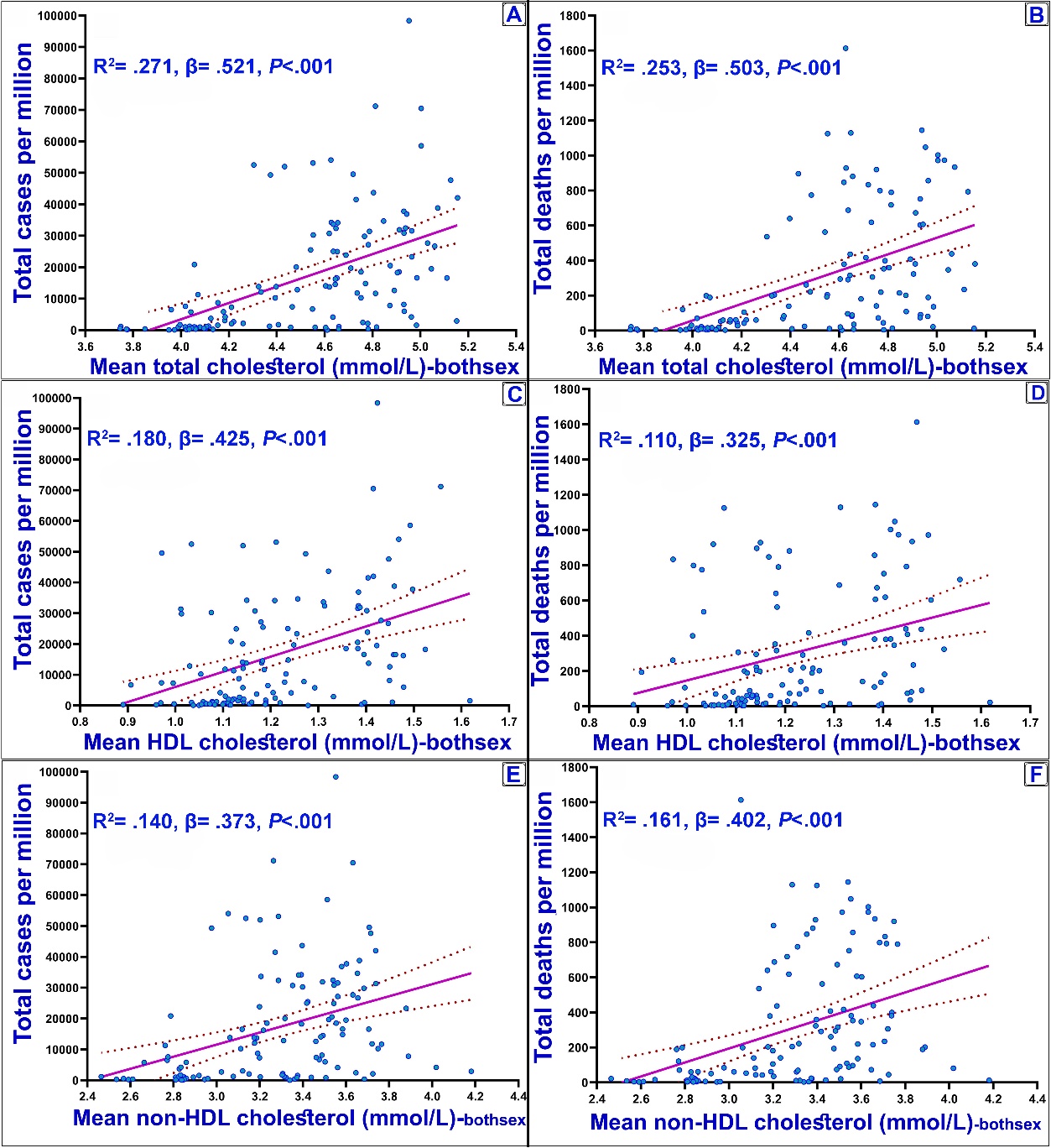


**Figure S1. Cases and deaths ratio of COVID-19 associated with cholesterol variables.** a&b: mean total cholesterol (mmol/L) (age-standardized both sex) reported in 2018 for evaluated countries; c&d: Mean non-HDL cholesterol (mmol/L) (age-standardized both sex); e&f: Mean HDL cholesterol (mmol/L) (age-standardized both sex). Best-fit lines (solid purple line) and the 95% confidence intervals of the best-fit line (dashed) by linear regression are indicated.
